# A User’s Guide to Golden Gate Cloning Methods and Standards

**DOI:** 10.1021/acssynbio.2c00355

**Published:** 2022-11-02

**Authors:** Jasmine E. Bird, Jon Marles-Wright, Andrea Giachino

**Affiliations:** † School of Computing, Faculty of Science Agriculture and Engineering, Newcastle University, Newcastle upon Tyne, NE1 7RU, United Kingdom; ‡ Biosciences Institute, Faculty of Medical Sciences, Newcastle University, Newcastle upon Tyne, NE2 4HH, United Kingdom; § School of Science, Engineering & Environment, University of Salford, Salford, M5 4NT, United Kingdom

**Keywords:** Golden Gate, MoClo, standards, cloning, restriction enzyme

## Abstract

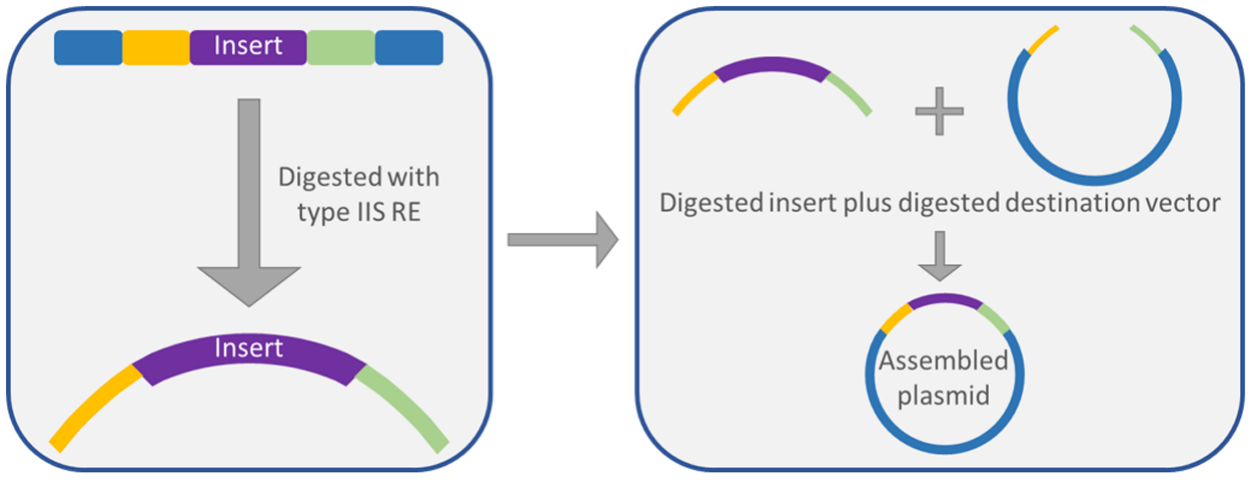

The continual demand for specialized molecular cloning techniques that suit a broad range of applications has driven the development of many different cloning strategies. One method that has gained significant traction is Golden Gate assembly, which achieves hierarchical assembly of DNA parts by utilizing Type IIS restriction enzymes to produce user-specified sticky ends on cut DNA fragments. This technique has been modularized and standardized, and includes different subfamilies of methods, the most widely adopted of which are the MoClo and Golden Braid standards. Moreover, specialized toolboxes tailored to specific applications or organisms are also available. Still, the quantity and range of assembly methods can constitute a barrier to adoption for new users, and even experienced scientists might find it difficult to discern which tools are best suited toward their goals. In this review, we provide a beginner-friendly guide to Golden Gate assembly, compare the different available standards, and detail the specific features and quirks of commonly used toolboxes. We also provide an update on the state-of-the-art in Golden Gate technology, discussing recent advances and challenges to inform existing users and promote standard practices.

## Introduction

Custom DNA constructs play a fundamental role in biological research as cheap, easy-to-manipulate carriers of genetic information.^1^ Their applications include targeted genome editing,^2^ the expression of recombinant proteins,^3^ the construction of synthetic gene circuits,^4^ artificial genomes,^5^ and cell-free biosynthesis.^6^ Given the low price of oligonucleotide synthesis, typically in the range of $0.10 per base,^7^ purchasing short DNA sequences from commercial suppliers is a ubiquitous practice in molecular biology research. Double stranded DNA fragments in the kilobase range are also readily available thanks to recent advances in DNA synthesis technology.

With the ability to acquire arbitrary synthetic DNA sequences quickly and cheaply, there is a need for methods to assemble these into larger, useful constructs.^7,8^ This is particularly required in the case of larger constructs, whose length is not suitable for chemical synthesis, and in the context of shuffling and optimization studies, in which the same genetic elements must be assembled in multiple ways. Popular methods for DNA assembly include exonuclease digestion-ligation (Gibson assembly),^9^ assembly PCR,^10^ and in vivo assembly, which exploits the DNA repair and homologous recombination abilities of a living chassis.^11^ One common drawback of these methods is that they only accept linearized DNA parts as substrates, and therefore depend on a time-consuming intermediate DNA purification step.

**Figure 1:**
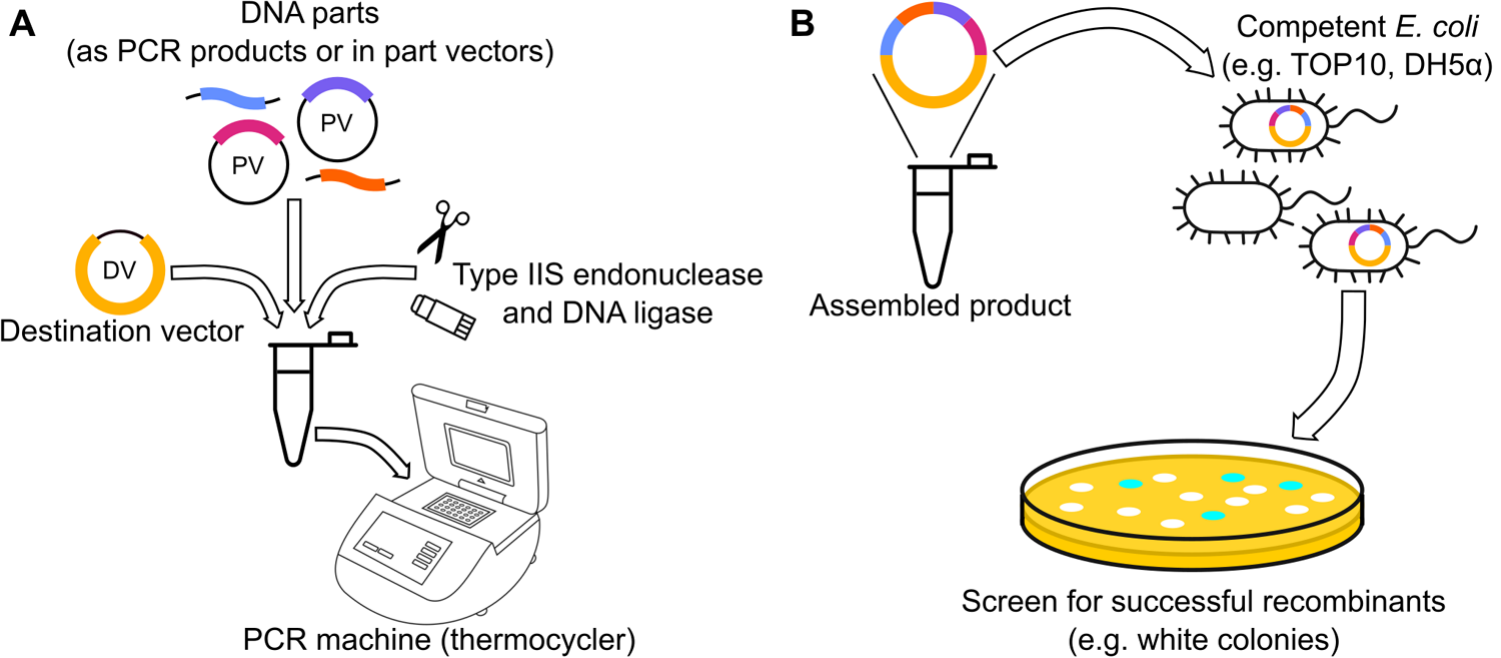
The two steps of Golden Gate assembly. (A) Individual parts are provided as plasmid-borne cargo or linear PCR products and mixed in a single tube together with the restriction enzyme (scissors) and the DNA ligase (glue); the tube is then inserted in the thermocycler for ligation. (B) The crude reaction mixture is transformed directly into the recipient *E. coli* strain and selected on Agar plates. No intermediate assembly steps are required, and only a single restriction enzyme is needed regardless of the number of assembly parts.

By contrast, one DNA assembly method that has gathered significant traction in the synthetic biology community is restriction enzyme-mediated assembly using Type IIS (shifted) endonucleases, also known as Golden Gate.^12^ This method can be used to combine large numbers of DNA parts in a one-pot assembly reaction, which can then be transformed directly in a recipient strain for selection and propagation (Figure 1). Importantly, Golden Gate cloning accepts both linear and circular DNA molecules as substrates. This makes it possible to create standardized libraries of assembly-ready parts in storage plasmids, which are easy to propagate, purify, and distribute. Moreover, it obviates the need to linearize and purify individual DNA parts ahead of assembly, providing a quicker, more facile alternative to other assembly methods such as Gibson assembly.

Crucially, because Golden Gate parts can be stored within plasmid vectors, they can be easily distributed to different laboratories: once a part is made, it can be propagated in cloning strains and easily shipped to collaborators, customers, and repositories. Libraries of Golden Gate-ready parts are now available for a variety of host organisms and projects, ranging from protein expression in bacteria to CRISPR/Cas genome editing in eukaryotes and protein localization to mitochondria and chloroplasts (Table 1).

**Table 1:** Complete List of Golden Gate Toolkits Following the Common Syntax^a^

name	availability	ref	content of kit	part plasmids marker	part plasmids out enzyme
MoClo Toolkit	Addgene toolkit 1000000044	(59)	Empty backbones for DNA part domestication and hierarchical MoClo assembly.	SpeR	BsaI
Orzaez Lab GoldenBraid 2.0 Kit	Addgene toolkit 1000000076	(49)	Destination vectors and assorted parts to get started with Golden Gate, specifically GoldenBraid. All vectors are binary *E. coli–A. tumefaciens* plasmids and compatible with plant synthetic biology.	CamR or AmpR	BsaI
MoClo Plant Parts Kit	Addgene toolkit 1000000047	(48)	A complete toolkit for plant transformation. Includes promoters, 5′ and 3′ UTRs, antigenic tags, subcellular localization signals, reporter CDSs, selectable markers, terminators, a suppressor of silencing, and two linkers.	SpeR	BsaI
CIDAR MoClo Parts Kit	Addgene toolkit 1000000059	(47)	Promoters, CDSs, and terminators for protein expression tuning in *E. coli*	AmpR^†^	BsaI
UbiGate Collection	Addgene toolkits 1000000144, 1000000145	(68)	*A. thaliana* ubiquitin, Ub-activating and -conjugating enzymes, and Ub ligases.	SpeR	BsaI
Yeast mitochondria toolkit	Addgene plasmids 101682–101712	(29)	GoldenBraid destination vectors including homology arms for genome integration in *S. cerevisiae*. Also includes yeast promoters, mitochondrial targeting signals, terminators and selection markers.	AmpR^†^	BsaI
FungalBraid	Addgene plasmids 119057, 119676–119678, 119705–119715	(69)	Fungal promoters, CDSs, terminators, and selection markers. Includes parts from *A. nidulans*, *N. crassa*, *C. heterostrophus*, *P. chrysogenum*, and *P. digitatum*.	CamR	BsaI
Mobius Assembly Vector Toolkit	Addgene toolkit 1000000134	(50)	An alternative set of Level 0 backbones using AarI as assembly enzyme, and chromogenic dropouts (*amilCP*, *spisPink*, and *sfGFP*) instead of *lacZα* to remove the need for X-Gal and IPTG. Also, an alternative method for hierarchical assembly based on 4-part cycling (as opposed to the 2-part cycling used in GoldenBraid).	CamR	BsaI
*Chlamydomonas reinhardtii* MoClo toolkit	From the Chlamydomonas resource center	(27)	Promoters, UTRs, terminators, tags, reporters, antibiotic resistance genes, and introns for use in green microalgae (*C. reinhardtii*, *C. ellipsoidea*, *Nannochloropsis sp.*, *D. salina* and so on).	SpeR	BsaI
MoClo Plant Parts II and Infrastructure Kit	Addgene toolkit 1000000135	(70)	An expanded set of parts, including for yeast two hybrid interaction or bacterial infection assays. Also includes destination vectors that are compatible with both Golden Gate and gateway cloning.	SpeR	BsaI
MIDAS: A Modular DNA Assembly System for Synthetic Biology	Addgene plasmids 108332–108341	(62)	A set of end-linkers that contain outward sites for hierarchical assembly: by including one in the assembly, it acts as a placeholder that can be replaced later by additional elements. This makes is possible to add new parts *within* an assembly (as opposed to at one end).	SpeR	BsaI
Loop assembly	From the corresponding author (Addgene submission pending)	(63)	Destination vectors for hierarchical assembly. A variation of the GoldenBraid method, which assembled four intermediate parts at each assembly step.	CamR	BsaI
CIDAR MoClo Extension, Volume I	Addgene toolkit 1000000161	(71)	More promoters, UTRs, CDSs, and terminators.	AmpR^†^	BsaI
MoChlo: Modular Cloning Chloroplast Toolbox	Addgene toolkit 1000000156	(72)	Chloroplast-specific genetic modules. Also includes destination vectors for tobacco and potato.	KanR	BsaI
CyanoGate Kit	Addgene toolkit 1000000146	(26)	DNA parts and acceptor vectors for integrative and episomal vectors in cyanobacteria.	SpeR	BsaI
Expanded CRISPR-associated (Cas) toolkit	Addgene plasmids 117520–117647	(73)	Cas nucleases from different bacterial species, together with engineered variants of Cas9. Also includes premade expression cassettes targeting genes in *A. thaliana* and *N. benthamiana*.	SpeR	BsaI
*Dictyostelium discoideum* GoldenBraid toolkit	From the corresponding author	(28)	DNA parts and binary backbones for amoeba synthetic biology.	CamR	BsaI
Parts for geminiviral expression vectors	Addgene plasmids 106207–106218	(74)	Parts for geminiviral expression vectors. Also an additional set of α backbones for GoldenBraid assemblies with more than 2 parts per cycle.	AmpR^†^	BsaI
MoClo CRISPR/Cas Toolkit for Plants	Addgene toolkit 1000000159	(75)	CRISPR/Cas nucleases and gRNA backbones.	SpeR or KanR	BsaI
uLoop: Universal Loop Assembly	From the corresponding author (Addgene submission pending)	(64)	An expansion of the loop assembly toolkit including binary destination vectors for diatoms, yeast, plants, and bacteria.	SpeR	BsaI
*Phaeodactylum tricornutum* uLoop toolkit	Addgene plasmids 154036–154043	(25)	Promoters and terminators for expression in diatoms.	SpeR	BsaI

a Toolkits are listed in order of publication and include accession numbers where applicable. All these toolkits follow the common syntax.^24^ For toolkits that do not follow the common syntax, see Table 2. The selection marker for part plasmids (SpeR: spectinomycin; CamR: chloramphenicol; AmpR: ampicillin; KanR: kanamycin) and the endonuclease needed to liberate DNA parts from the part plasmids are also included. Note that toolkits whose part plasmid marker is AmpR (marked with ^†^) are not compatible with the MoClo pipeline, which uses AmpR as the selection marker of destination vectors (Level 1). Similarly, the preferred destination vectors in GoldenBraid (α vectors) carry KanR and are therefore incompatible with KanR part plasmids; however, GoldenBraid also provides SpeR destination vectors (Ω vectors), which are still compatible with KanR part plasmids. Also, note that all toolkits in the table use BsaI as the “out” endonuclease for part plasmids.

Despite its many advantages, the uptake of Golden Gate-based cloning has been slow outside of the synthetic biology community.^13^ Furthermore, research on how to further optimize the method is still ongoing^14−16^ and so is the definition of a shared, accepted standard (or “syntax”) for designing Golden Gate-compatible DNA parts.^17^ The sheer number of Golden Gate-related publications, with their different methods, syntax, and part libraries is a major obstacle to adoption by new users, and even existing users might struggle keeping up with recent developments in the field.^18^ In fact, although the core Golden Gate method has been thoroughly reviewed,^19,20^ few studies have addressed the breadth of different methods and tools that make up the Golden Gate family.

In this critical review, we summarize the current state of the art in Golden Gate-based cloning, highlighting the most accepted standards, but also drawing attention to recent developments and competing variants. Furthermore, we discuss the ongoing challenges and opportunities in optimizing the Golden Gate core methodology. As we show in the present work, there is no “Swiss knife” Golden Gate method that can fit all cloning purposes; instead, the Golden Gate family includes multiple assembly strategies for different circumstances. By providing the synthetic biology community with a comprehensive guide to current Golden Gate assembly methods, we hope to reduce the barrier to adoption of Golden Gate by new users, as well as providing suggestions for existing users who wish to update their workflows to the most recent standards.

## The Golden Gate Core Method

The core Golden Gate method, which was recently thoroughly reviewed,^19^ has remained essentially unchanged since its first proposal from Marillonnet and co-workers in 2008^12^ building on previous work by Fromme and Klingenspor^21^ and Kotera and Nagai.^22^

**Figure 2:**
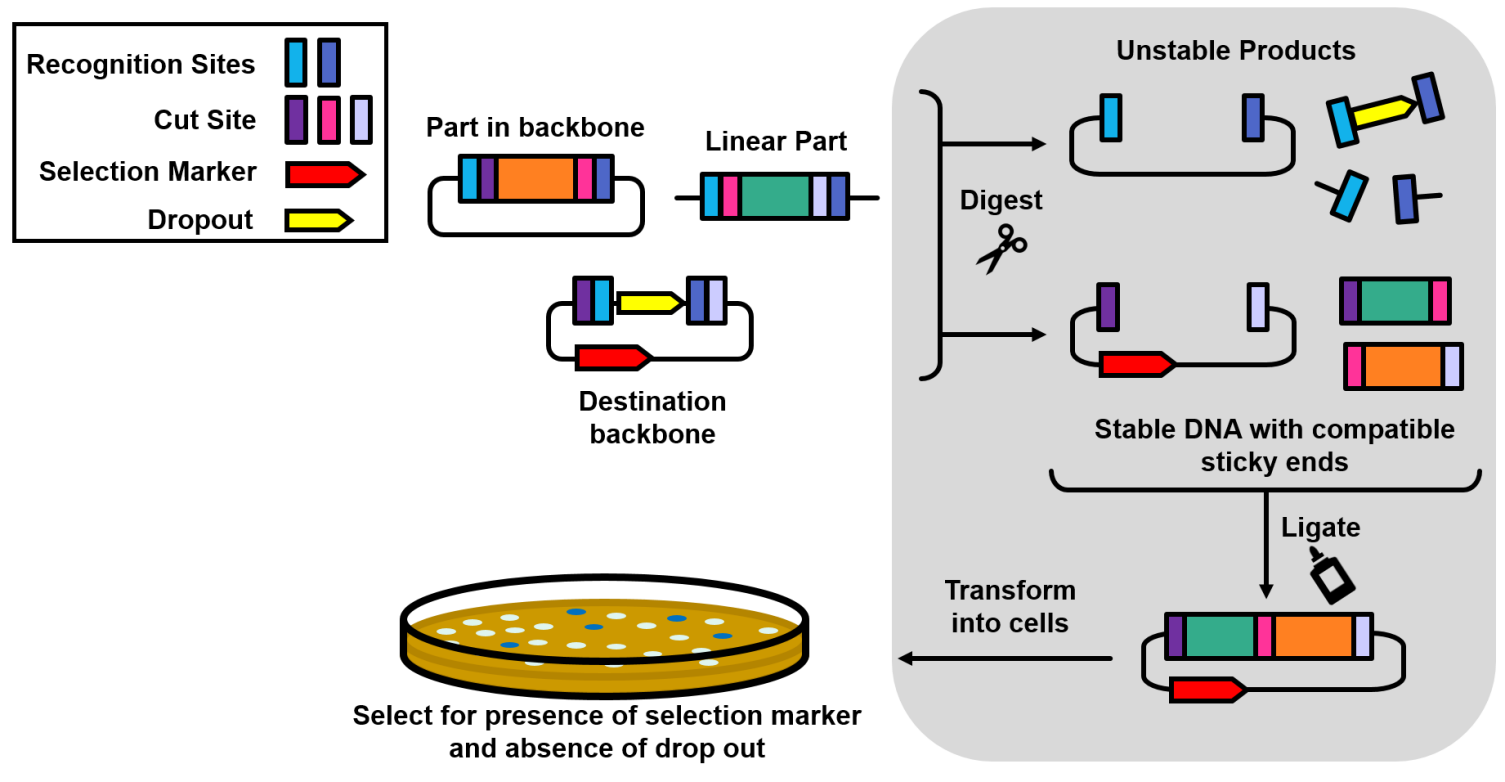
Enrichment of the target product. A Type IIS recognition site contains two separate elements: the recognition site (blue) where the enzyme binds DNA, and the cut site (white) where the enzyme cuts the DNA double helix. During Golden Gate assembly, all DNA molecules that contain recognition sites can be digested multiple times, and are therefore lost during assembly. Only parts that contain the cut sites, but not the recognition sites, will assemble into the final product.

Briefly, Golden Gate cloning (and its many derivative methods) assemble DNA molecules through the annealing of ssDNA sticky ends, which are generated by a Type IIS restriction endonuclease. These restriction enzymes cut DNA at a fixed distance to their recognition sequence (the restriction site is shifted), meaning that their recognition sequence only determines *where* the endonuclease will cleave DNA, but not at *what* bases. A single Type IIS endonuclease can generate ssDNA sticky ends with arbitrary nucleotide sequences by simply placing an endonuclease recognition sequence at the right distance from the target cutting site. A proper design of the position and orientation of cleavage sites also ensures that the recognition sequences will not be retained in the final construct, which is therefore resistant to further digestion (Figure 2).^12,19^

As a consequence, Golden Gate assembly only requires a single endonuclease, no matter the number of sticky ends to be used in the assembly: successful, high-yield assemblies of up to 35 parts have been reported.^15,23^ Moreover, Golden Gate achieves unparalleled yield and fidelity when compared to other methods, as any unwanted side product is converted back into reagents and only the stable final product accumulates in the reaction mixture. Since the final product is stable, the DNA ligase can be mixed together with the endonuclease in the same reaction mixture, resulting in a one-pot reaction that can easily be performed in a benchtop thermocycler.^19^ No intermediate DNA purification step is required, and the crude reaction mixture can be directly transformed in a recipient strain (such as *E. coli* TOP10) for product selection and subsequent propagation.

## Fusion Site Design

**Figure 3:**
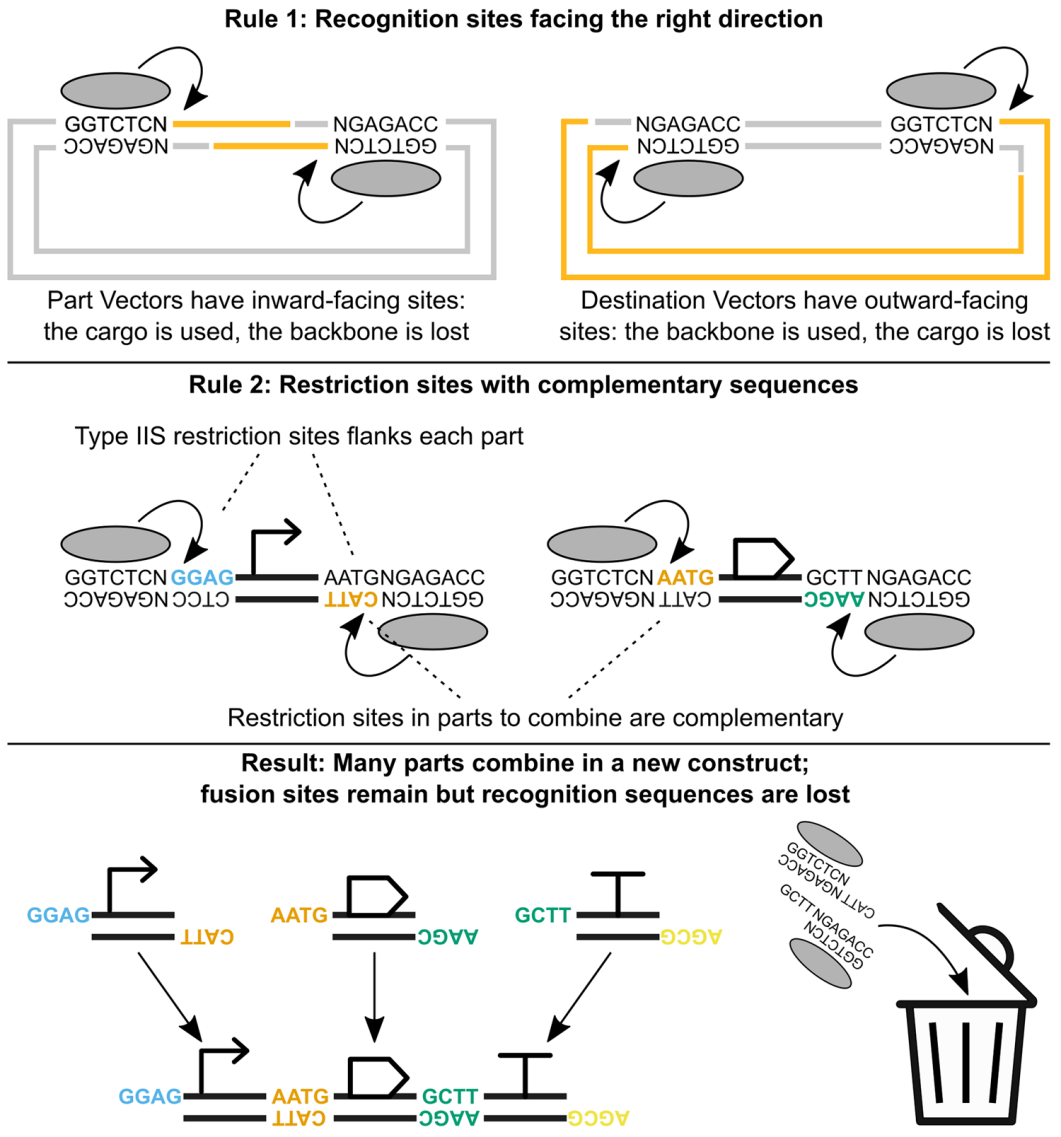
Proper design of restriction sites. For Golden Gate assembly to be successful, all part vectors must have inward-facing Type IIS sites, so that the enzyme recognition sequence is in the backbone (not the part); at the same time, destination vectors must have outward-facing sites, with the recognition sequence in the part (not the backbone). Also, the sequence of the fusion sites must be chosen so that parts that will be adjacent in the final assembly have complementary fusion sites. Clearly, the destination vector must have sites complementary to the *first* and the *last* fusion sites in the assembly, so that the entire construct will ligate into the destination vector.

The most important concept in Golden Gate assembly is the “proper design” of endonuclease restriction sites (Figure 3), which is thoroughly reviewed elsewhere.^20^ These restriction sites make it possible to extract DNA parts from part vectors and insert the assembled construct into destination vectors.

Briefly, the restriction site of a Type IIS endonuclease comprises two parts: the recognition sequence, where the enzyme binds DNA; and the cleavage site, where the enzyme cuts the DNA double helix. For example, the restriction site of the BsaI endonuclease is GGTCTCN^▼^NNNN_▲_, which includes a 6-nucleotide recognition sequence (GGTCTC), a 1-nucleotide spacer, and a 4-nucleotide cleavage site (^▼^NNNN_▲_), which is also the fusion site used in the assembly. For example, a DNA part beginning with a GGTCTCN^▼^AATG_▲_ site will ligate with a part ending with a ^▼^AATG_▲_NGAGACC site (note that NGAGACC is the reverse complement of GGTCTCN). Golden Gate parts that must be digested with a certain restriction endonuclease must also lack internal recognition sites for that endonuclease; the same applies to Golden Gate destination vectors, which should contain no additional recognition sites for the chosen endonuclease(s).^19,20^

Importantly, it is recommended that parts of the same type, such as different ribosome binding sites, should be flanked by the same pair of fusion sites. This way, they can all be ligated in the same position (usually between a promoter and a protein coding sequence), and can be easily exchanged between laboratories, as parts made by one research group will also work for all other groups following the same rules for DNA part constructions (also called a “syntax”). Conversely, if two groups or toolkits follow different syntaxes, their parts will not be compatible with each other, requiring further rounds of domestication to be ported between different standards.^19^

The most widely accepted syntax for Golden Gate assembly is the so-called “common syntax”.^17,24^ This standard set of fusion sites was originally defined in the plant and bacterial synthetic biology communities, but has since been applied to other organisms, such as diatoms,^25^ cyanobacteria,^26^ algae,^27^ amoebae,^28^ and yeast.^29^ A list of existing toolkits adopting the common syntax is provided in Table 1, and a comprehensive guide on how to choose fusion sites according to the common syntax is also available in the literature.^24^

Despite the widespread adoption of the common syntax, other standards are also used in certain laboratories (Table 2). Many groups working with plant transgenes use the syntax of Lohmann and co-workers,^30^ which also has its own toolkits.^31,32^ The same applies to the yeast syntax of Dueber and co-workers^33−37^ and the fungus syntax of Sauer and co-workers.^38−40^ Meanwhile, certain research groups use their own syntax, which is mainly expanded internally: see, for example, refs (41−43) and (44−46).

**Table 2:** Alternative Golden Gate Methods That Do Not Follow the Common Syntax^a^

assembly family	reference	content of kit	expansions
GreenGate	(30)	Parts for plant transgenesis.	(31,32)
Modular Plant Toolkit	(41)	An alternative assembly system with an additional slot after the terminator part for miscellaneous gadgets (e.g., selection markers or origins of replication for binary plasmids).	(42,43)
Mammalian MoClo	(76)	Parts for mammalian synthetic biology, focusing on the construction of *att* site-based integration vectors. It also includes a variety of insulator parts.	
Modular Yeast Toolkit	(33)	Yeast markers and origins.	(34−37)
YeastFab	(77)	Parts for *S. cerevisiae*.	
Yeast Golden Gate	(78)	Parts for *S. cerevisiae*.	
EcoFlex	(65)	Parts for *E. coli*.	
*Yarrowia* Golden Gate	(44)	An alternative assembly system to assemble three transcriptional units at the same time.	(45,46)
GoldenMOCS	(38)	Parts for gene integration in fungus.	(39,40)
MoPET	(79)	Parts for protein expression, particularly signal peptides, end tags, and flexible linkers.	
EMMA	(80)	Parts for mammalian synthetic biology, focusing on customizing the expression cassette and the selection marker at the same time.	
COSPLAY	(54)	Parts for *S. cerevisiae*.	
MTK	(81)	Parts for mammalian synthetic biology.	
TrichoGate	(57)	Parts for *Trichoderma*.	
GoldenBac	(82)	Parts for baculovirus expression vectors.	

a The assembly techniques in this list are alternatives to the more common MoClo, GoldenBraid, and similar methods that follow the common syntax. Each toolkit constitutes a separate Golden Gate family, which is not compatible with the others, nor with the common syntax group. Subsequent expansions of each family are also included in the “expansion” column.

It is strongly recommended that new users should use whatever syntax is the most accepted in their scientific community. If a local syntax has not been established, the common syntax should be preferred,^24^ as it is already shared by many laboratories worldwide and has the largest number of available toolkits.

## The Destination Vector

**Figure 4:**
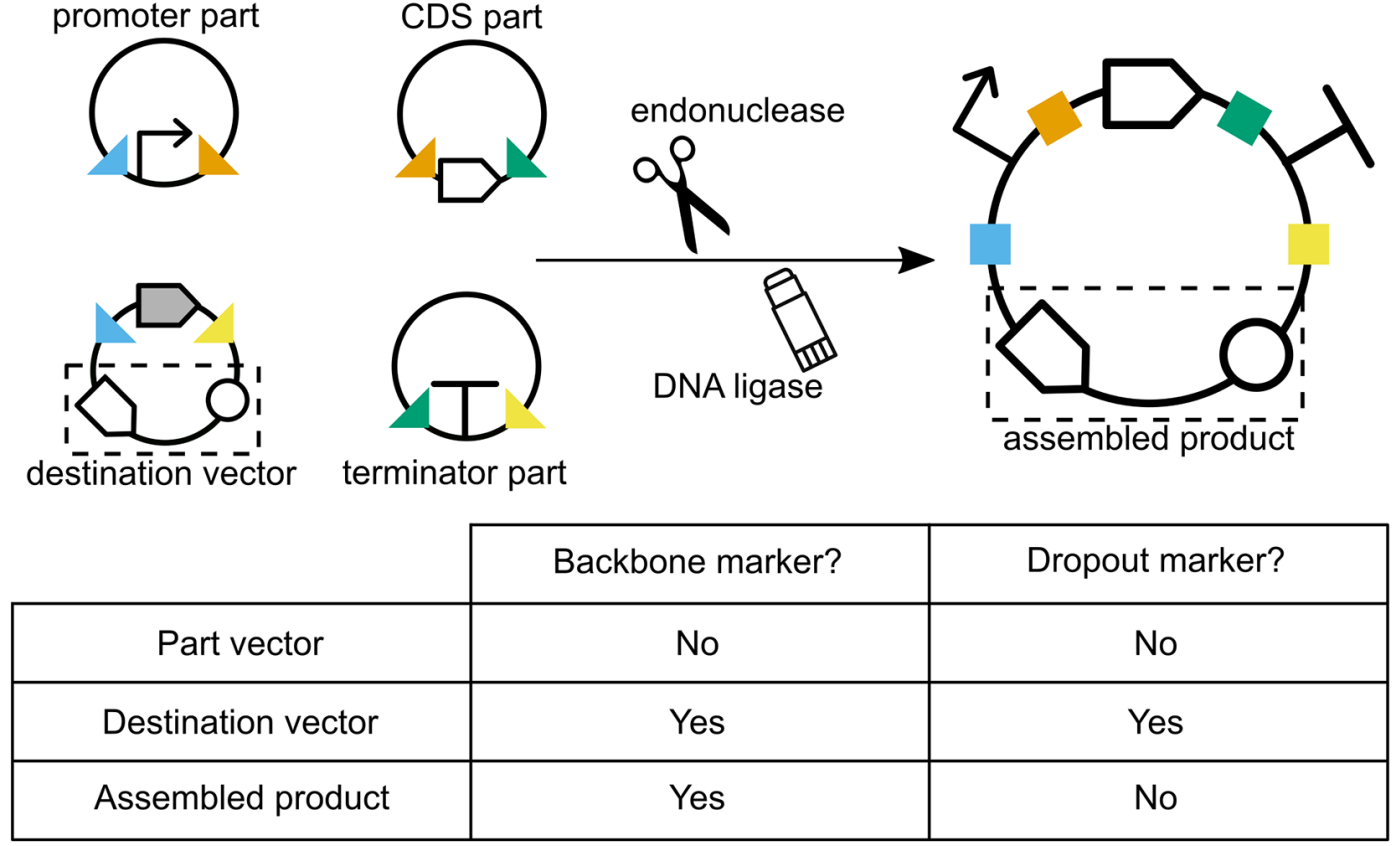
Destination vectors as acceptor backbones. The destination vector of a Golden Gate assembly provides the origin or replication and selection marker of the final construct (dashed box). Note that the fusion sites of the destination vector (<>) appear in the opposite orientation compared to those of the part vectors (><). The cargo of the destination vector (gray CDS) is a dropout marker, which is lost during the assembly. Therefore, the final construct can be isolated by combining positive selection for the backbone marker (typically antibiotic resistance), and negative screening against the dropout marker (usually a visual marker such as *lacZα*).

Another important factor in Golden Gate assembly is the choice of a proper destination vector. This contributes the acceptor backbone to the final assembly, and normally contains (i) the plasmid origin of replication that is desired for the final product; (ii) a selectable marker, such as a gene providing antibiotic resistance; and (iii) a drop-out marker, which is located between the endonuclease recognition sequences and is replaced by the assembled part upon successful ligation (Figure 4). In most cases, users can source destination vectors from published toolkits^47−49^ and do not need to design their own.

Crucially, the destination vector of an assembly must carry a different screenable marker compared to all part vectors in the assembly, to ensure that the only the final product (which has the destination vector’s backbone) will be retained in the selection step. For example, if part vectors carry a gene conferring resistance to ampicillin (a β-lactam antibiotic), the backbone of the destination vectors could confer resistance to kanamycin, chloramphenicol, or any non-β-lactam antibiotic. One could also use part vectors with different antibiotic resistances, as long as the destination vector has a different selection marker than *each* part vector. As an example, one could use a kanamycin-resistant destination vector with ampicillin-resistant part vectors, chloramphenicol-resistant part vectors, or a combination of both.

In addition to the selection marker and the endonuclease recognition sites, Golden Gate destination vectors also carry a counter-screenable gene as the dropout cargo, which is lost upon successful ligation. The dropout gene commonly encodes a lethal phenotype (such as *ccdB*) or allows for visual screening (such as a *lacZα* fragment for blue-white screening in a strain expressing *lacZω*). Other dropouts such as *amilCP*, *spisPink*, GFP, and others can also be used.^50^ This makes it possible to select colonies that contain the assembled product (which lacks the dropout) and discard those that contain undigested destination vectors (which still have the dropout).

The choice of destination vector also influences part domestication. For example, if a DNA part contains an internal BsaI site, it cannot be assembled in a BsaI-based destination vector. It is now considered best practice to remove internal sites for BbsI, BsaI, and BsmBI during part domestication,^19,20^ since these enzymes are used by the most widespread assembly methods (MoClo uses BbsI and BsaI, and GoldenBraid uses BsmBI and BsaI). Still, “illegal” internal sites are sometimes encountered in older toolkits, and it is always worth checking parts for compatibility with the intended restriction enzymes.

Finally, in some cases, a user may want to design their own destination vectors, for example if they work with an organism for which no toolkit is available, or because they need specific markers, origins or replication, or other gadgets that are not included in published backbones. When designing new destination vectors, the best starting point is the Bacterial Expression Vector Archive (BEVA) toolbox of Poole and co-workers^51^ (AddGene plasmids 113979–14002), which defines a convenient standard for building destination vectors with custom selection markers, origins of replication and transfer, and cloning sites. Alternatively, Valenzuela-Ortega and French have proposed a different standard of SEVA-compatible Golden Gate destination vectors, called Joint Universal Modular Plasmids (JUMP), which follow the SEVA 3.1 standard and include cloning sites for BioBrick assembly with AarI and BbsI^52,53^ (Addgene plasmids 126956–127051). While the BEVA toolbox is more suitable for assembling destination vectors de novo, JUMP is designed to make changes to existing destination vectors. As standardization becomes more and more important in synthetic biology,^18^ we suggest that these methods should be regarded with particular interest.

## Hierarchical Assembly

Another strength of Golden Gate based methods is the ability to perform hierarchical assembly, that is, to reuse assembled products as DNA parts for subsequent assembly steps. The most common example of this is the assembly of a genetic circuit from individual transcriptional units: for practical reasons, it is often advantageous to assemble each transcriptional unit separately (stage 1 of the assembly) and then combine the separate transcriptional units into a single construct (stage 2 of the assembly). Another example is when a transcriptional unit must first be assembled individually, and then combined with additional modules such as helper genes,^26,38^ selection markers,^30,33,54−56^ replication origins for different hosts,^33,54^ centromeres,^54,56^ targeting sequences for genomic integration,^26,38,39,44,55,57,58^ or origins of transfer (*oriT*).

**Figure 5:**
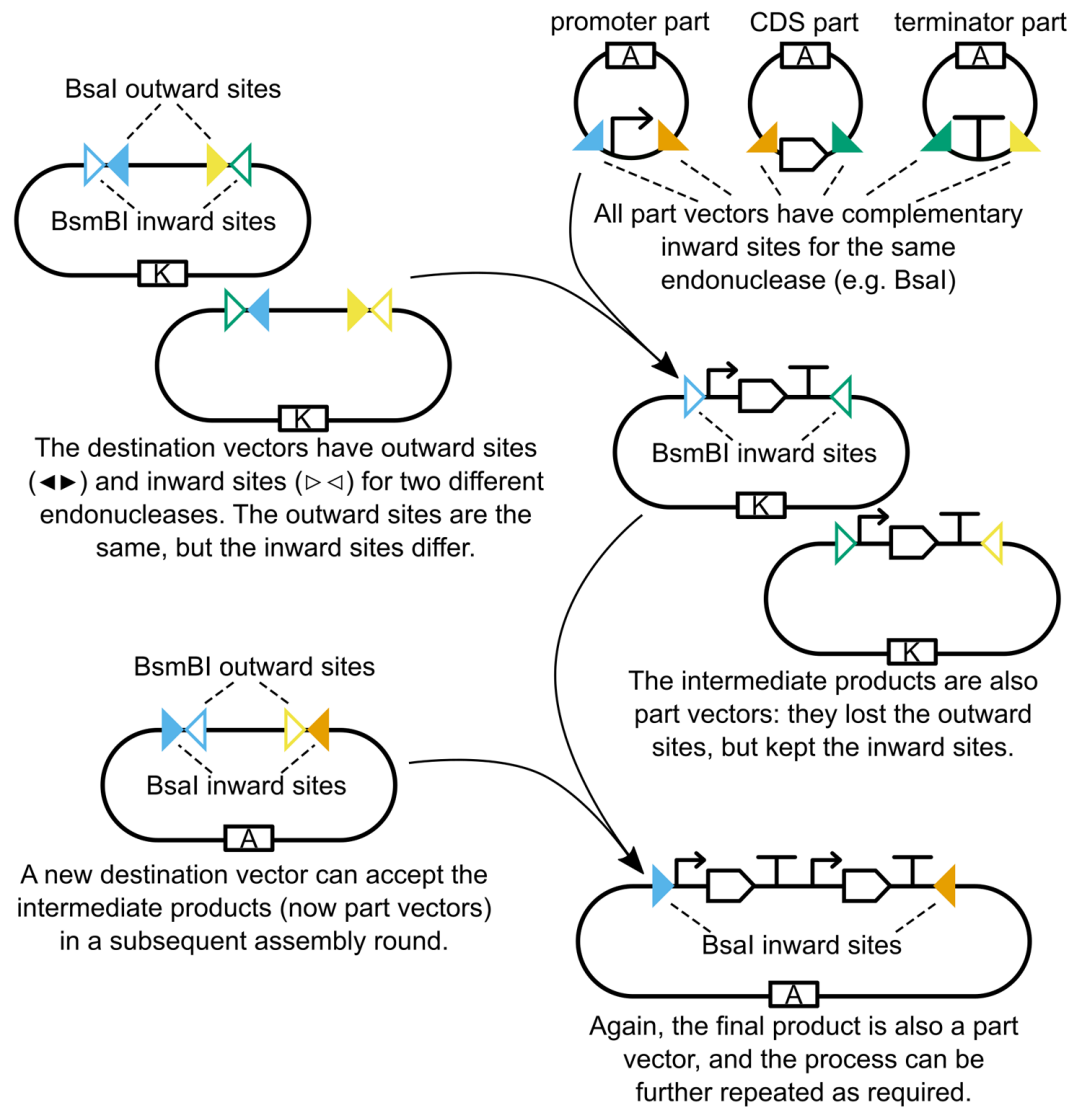
Multilevel hierarchical assembly. If a destination vector contains inward sites in addition to outward sites, the inward sites will be retained in the final assembly and can then be used to excise the product as if it was a part. In this example, both destination vectors have the same outward-facing sites (blue<>yellow), but different inward-facing sites (respectively blue><green and green><yellow). Consequently, the intermediate products are also part vectors, and share a complementary fusion site (green). The intermediate parts can be assembled in a new destination vector, resulting in a final product that is also a part vector. Note that each assembly step uses a different endonuclease (here BsaI and BsmBI) and resistance marker (here ampicillin and kanamycin).

Even though hierarchical assembly is one of the core strengths of Golden Gate, it also adds complexity to the method. Just like any other Golden Gate assembly, a hierarchical assembly relies on properly designed cleavage sites, as reviewed elsewhere.^12,19,20^ Specifically, it requires destination vectors with two pairs of recognition sites, each recognized by a different endonuclease (Figure 5): one to insert the intermediate construct (e.g., a transcriptional unit) into the destination vector, and another one to liberate the assembled construct for further assembly rounds (e.g., combining two transcriptional units together). It also requires at least two restriction endonucleases (for example BsaI and BsmBI) and two selection markers (for example kanamycin and spectinomycin) since these must be alternated between different assembly steps.

In addition to the additional resources required, multistep assembly also requires more extensive planning. Each assembly step must be planned, so that all intermediate parts at each assembly step will have compatible fusion sites with each other; otherwise, one could accidentally end up with two parts that cannot be further combined. Moreover, there are many different methods of hierarchical assembly, each requiring a different set of destination vectors, and certain Golden Gate toolboxes are not compatible with hierarchical assembly at all.

Currently, the most widespread methods for hierarchical assembly are the MoClo method of Marillonnet and co-workers^48,59^ and the GoldenBraid method of Orzaez and co-workers.^49,60,61^ The main differences between these methods are the number of destination vectors required, and the number of intermediate parts that can be combined at each assembly step. While the GoldenBraid method only requires a minimal set of destination vectors (8 in total), it only assembles two intermediate parts at each assembly cycle. By contrast, the MoClo method can assemble a larger number of intermediate parts but requires a much larger suite of vectors and also dedicated DNA end-linkers to achieve the additional flexibility.

Importantly, both MoClo and GoldenBraid are fully compatible with the standard syntax,^24^ making them also partially compatible with each other. DNA parts can be easily transferred between systems using intermediate destination vectors that contain recognition sequences for many different enzymes. For example, part vectors in the GoldenBraid standard contain inward-facing sites for two different enzymes, BsaI and BtgZI, which cut DNA in the same place.^49^ If, for whatever reason, BsaI cannot be used in the assembly, BtgZI can be used instead. Mascher and co-workers have also devised an expanded architecture for inward facing sites that includes recognition sequences for five different Type IIS endonucleases (AarI, BtgZI, BbsI, BsaI, BsmBI), making it possible to transfer DNA parts across multiple different standards.^55^

In addition to MoClo and GoldenBraid, there are also other, less widespread methods for hierarchical assembly. The MIDAS system^62^ operates by adding a dropout placeholder to the intermediate assembly, which can be replaced later by the addition of a different part. Meanwhile, Loop assembly is a slightly tweaked derivative of GoldenBraid which uses four parts at a time instead of two.^63,64^ Although these methods might be useful for certain projects, they are less widely used than MoClo or GoldenBraid. Another way to achieve hierarchical assembly is by using destination vectors with more than one entry site, such as those provided in the EcoFlex and JUMP toolkits:^53,65^ this way, a first, complete part can be assembled first into the destination vector (potentially as part of a MoClo or GoldenBraid pipeline) and a second part can subsequently be added into the same vector using a different restriction site.

## Functionally Scarless Assembly

Due to the design of parts and vectors used in Golden Gate assembly methods, the recognition sequences for restriction enzymes are not retained in the resulting assembly products. Therefore, this assembly method can be considered functionally scarless in some cases. Specifically, Golden Gate fusion sites can be designed to correspond to nucleotide sequences that would normally be present in the final product. For example, the Standard Syntax uses the 4-nucleotide AATG fusion site to link together promoter parts with coding sequence parts, since this fusion site already contains an ATG start codon which would normally be required at the 5′ end of coding sequence. Similarly, standard fusion sites that are part of the coding sequence of fusion proteins (for example, linking a coding sequence part with a N-terminal tag part) are designed to include a codon for a small amino acid, such as glycine or serine, that is commonly found in linker sequences. The resulting open reading frame does not retain the restriction enzyme’s recognition sequences, making the assembly functionally scarless.

Even though the nucleotide fusion site is not by itself an assembly scar, it must still be considered in order to correctly design Golden Gate parts, especially in the case of protein fusion constructs. When designing a protein fusion, the 4-nucleotide overhangs generated by many Type IIS restriction enzymes (notably BsaI, BbsI, and BsmBI) should normally be flanked by two extra nucleotides to ensure the correct reading frame (six nucleotides instead of four). This adds an extra layer of complexity to part design: if a user forgets to add the two extra nucleotides to a part (or remove one, which also restores the reading frame), they could end up with an accidental frameshift in their final fusion construct. While this small adjustment may come as second nature for long-term Golden Gate users, it may also be especially daunting for new users.

To remove the potential for error, a few assembly standards utilize 3-nucleotide fusion sites, which naturally conserve the reading frame, instead of the traditional 4-nucleotide fusion sites. This is made possible by using different Type IIS restriction endonucleases, such as SapI and EarI, which cut DNA over a trinucleotide overhang, instead of the tetranucleotide overhang generated by BsaI, BbsI, and BsmBI. An example of these methods is Start–Stop Assembly,^16^ which makes it possible to assemble transcriptional units without introducing accidental frameshifts. Even though a 3-nucleotide syntax can draw from a smaller pool of possible fusion sites (32 possible trinucleotides as opposed to 128 tetranucleotides), Lohmann and co-workers have already identified a set of 13 trinucleotides that can be used in the same assembly while retaining >90% fidelity,^23^ which is comparable with that achieved by 4-nucleotide syntaxes. Importantly, this is more than the 11 orthogonal fusion sites required by the common syntax,^24^ meaning that a full conversion of the common syntax into 3-nucleotide space is possible. Indeed, Yang and co-workers have provided a toolkit for a full conversion of GoldenBraid into a 3-nucleotide syntax, using SapI and EarI as restriction enzymes.^66^

Overall, 3-nucleotide syntaxes appear poised to compete with existing methods, as they result in a simpler part design: because one does not need to account for potential frameshifts, it is easier to design parts “correctly” when using a 3-nucleotide syntax. On the other hand, the use of BsaI, BbsI, and BsmBI is widely accepted in the synthetic biology community and has been further cemented by the introduction of the common syntax.^24^ This creates a barrier to further adoption of SapI and EarI, given that existing collections of part plasmids would require extensive redomestication to be imported into a 3-nucleotide syntax. As for any other standard, the biggest challenge that 3-nucleotide syntaxes must face is successful uptake by both new and existing users.^13^

## Combining Parts from Different Toolkits

As discussed above, there are currently a range of different standards for Golden Gate assembly. These variations of the method differ in the choice of fusion sites, restriction endonucleases used, and resistance markers for the destination vectors, as well as on whether and how they can be used for hierarchical assembly. In many cases, users will be most familiar with toolkits belonging to their same family: for example, GoldenBraid users will typically work with GoldenBraid toolkits. However, users might also want to access parts from a toolkit in a different family at some point. When combining parts from different toolkits, some additional factors must be considered to ensure a successful assembly.

First, the use of a similar part syntax (typically the common syntax^24^) should mean that DNA parts will be cross compatible between standards; however, even parts designed according to the common syntax may still contain internal recognition sequences for restriction endonucleases used in certain assembly standards. For example, MoClo uses the endonucleases BsaI, BbsI, and optionally BsmBI, meaning that parts designed for the MoClo standard do not contain internal recognition sites for these enzymes. GoldenBraid, on the other hand, only uses BsaI and BsmBI, and unless GoldenBraid parts are explicitly designed to exclude BbsI recognition sequences, those parts will not be compatible with MoClo. Similar considerations apply to any pairs of assembly standards that do not share the same endonucleases. Therefore, if a user wishes for their parts to be cross-compatible, they must be careful to remove a range of potential recognition sites when designing or domesticating a part.

Additionally, users should also consider how parts will be transferred from one standard to the other, as the antibiotic selection markers used in the part and destination vectors often differ between toolkits (see Table 1). For example, if a toolkit contains parts that are stored in kanamycin-resistant part vectors, those parts cannot be assembled into a kanamycin-resistant destination vector, since the resistance markers for part and destination vectors must differ (see Hierarchical Assembly, earlier). When importing Golden Gate parts from a toolkit to another, it is often advisable to redomesticate them into part vectors from the new toolkit if these contain a different selection marker than the original one. Until the time when a unified standard for Golden Gate is established, including both restriction endonucleases and selection markers, such cross-compatibility barriers will remain between parts belonging to different toolkit families.

## Conclusions and Perspectives

Golden Gate DNA assembly methods represent a powerful toolkit for projects where the same DNA parts will be reused multiple times, either because they will be put together in different ways as part of a combinatorial library, or because they will be subsequently joined in hierarchical assemblies. Since the publication of the original Golden Gate method in 2008^12^ and the MoClo and GoldenBraid standards in 2011,^59,60^ there has been a proliferation of part toolkits with varying levels of standardization. Each of these toolkits shares the same core protocol which remains broadly unchanged,^19^ but may include variations for the assembly of constructs of increasing complexity. Given the utility and adaptability of Golden Gate methods, it is no surprise that there is a great deal of ongoing research and development of new toolkits of parts for different host chassis and purposes. Despite the efforts to introduce a core part syntax, starting with the common syntax for plant and microbial synthetic biology,^24^ many methods have expanded or deviated from this.

As with most methods in molecular biology, the strongest factor influencing the method used for a particular project is the historic adoption in the laboratory the project is being performed in, or in a collaborator’s laboratory. In this case, the “best” method for a certain user is simply the one that is used in the user’s community. A note of caution should be sounded for the user to reflect on the final assemblies and outcomes required of a particular project, ideally at the start of the project and not after significant time and effort has been expended designing and building DNA constructs. We hope that this review provides both the novice and expert molecular biologist with an overview of the current state of the art and choice of methods and toolkits available for their projects.

Looking ahead to the future, a major threat to the more widespread adoption of Golden Gate methods, notwithstanding the decades of accreted constructs and expertise in molecular biology laboratories, is the rapid development of new methods and toolkits. This apparent paradox is common to all standards and nicely parodied in a widely cited XKCD cartoon (https://xkcd.com/927/). Indeed, even during the writing of this review, a new preprint offering another Golden Gate standard was posted to the bioRxiv.^67^ Given the number of different scientists, the vast number of molecular biology projects, and the infinite number of potential DNA constructs, there will never be a one-size fits all approach to DNA assembly. To tackle this issue, it is important that the synthetic biology community balances the requirement for accepted standards while at the same time adopting key innovations as they are introduced. Frequent communication between different groups of tool makers and users will be pivotal in ensuring that the most appropriate tools are adopted and are used to realize the infinite diversity of imaginable DNA constructs.
